# Rapid Screening and Identification of Diterpenoids in *Tinospora sinensis* Based on High-Performance Liquid Chromatography Coupled with Linear Ion Trap-Orbitrap Mass Spectrometry

**DOI:** 10.3390/molecules22060912

**Published:** 2017-05-31

**Authors:** Lu-Lu Xu, Feng-Xia Guo, Sen-Sen Chi, Zi-Jian Wang, Yan-Yan Jiang, Bin Liu, Jia-Yu Zhang

**Affiliations:** 1 School of Chinese Pharmacy, Beijing University of Chinese Medicine, Beijing 100029, China; xll@bucm.edu.cn (L.-L.X.); gfx521713@163.com (F.-X.G.); m17801085298@163.com (S.-S.C.); jyyjm1129@163.com (Y.-Y.J.); 2 Beijing Research Institution of Chinese Medicine, Beijing University of Chinese Medicine, Beijing 100029, China; helloffiresilver@gmail.com

**Keywords:** HPLC-LTQ-Orbitrap, diterpenoids, characteristic fragmentation pathways, *Tinospora sinensis*

## Abstract

Diterpenoids are considered the major active compounds in *Tinospora sinensis* in virtue of their special structures and activities. Herein, an analytical method was developed for rapid screening and identification of diterpenoids in *T. sinensis* using high-performmance liquid chromatography coupled with linear ion trap-Orbitrap mass spectrometry (HPLC-LTQ-Orbitrap) in negative ion mode. Two diterpenoid reference standards were first analyzed to obtain their characteristic ESI-MS/MS fragmentation patterns. Then, based on the extracted ion chromatogram (EIC) data-mining method and characteristic fragmentation pathways analysis, diterpenoids in *T. sinensis* were rapidly screened and identified. After that, an important parameter, Clog *P*, was adopted to discriminate between the isomers of diterpenoids. As a result, 63 diterpenoids were characterized from the extract of *T. sinensis*, including 10 diterpenoids and 53 diterpenoid glycosides. Among them, 15 compounds were tentatively identified as new compounds. Finally, target isolation of one diterpenoid glycoside named tinosineside A was performed based on the obtained results, which further confirmed the deduced fragmentation patterns and identified diterpenoid profile in *T. sinensis*. The results demonstrated that the established method could be a rapid, effective analytical tool for screening and characterization of diterpenoids in the complex systems of natural medicines.

## 1. Introduction

*Tinospora sinensis* (Kuanjinteng in Chinese) is commonly known as ‘Gurch’ and belongs to the family Menispermaceae, which is mainly distributed in the tropical parts of the eastern hemisphere. It has been traditionally used as folk medicine for treating debility, dyspepsia, rheumatism, gonorrhea, fever, inflammation, syphilis, ulcer, bronchitis, jaundice, urinary disease, skin disease and liver disease [1,2,3]. Previous phytochemical investigations have discovered that this species contains diterpenoids, alkaloids, flavonoids, lignans, triterpenes, amino acids, and so on. These constituents were reported to exert anti-inflammatory, immunomodulatory, antidiabetic, antitumor, antiadhesive, antiviral, anti-infective, antioxidant, antimutagenic, hematopoietic activities, etc. [4,5,6,7,8]. To our best knowledge, few reports on the systematic analysis of the diterpenoids in *T. sinensis* are available until now.

Recently, with the development of various data acquisition methods, high-resolution mass spectrometry (HRMS), especially linear ion trap-Orbitrap mass spectrometer (LTQ-Orbitrap), has exhibited excellent performance in the detection of targeted mixture constituents owing to its high speed and detection sensitivity [9,10]. The hybrid linear ion trap-Orbitrap mass spectrometer (LTQ-Orbitrap MS) combines high trapping capacity and MS^n^ scanning function of a linear ion trap together with accurate mass measurements within 3 ppm. The resolution power (up to 100,000) over a wider dynamic range is superior to that of many other mass spectrometers [11,12,13]. In the meantime, the combined application of tandem mass spectrometry for identifying the complicated constituents in traditional Chinese medicines (TCMs) could produce a large quantity of information data, such as molecular weights, elemental compositions, fragmentation patterns of multiple-stage, etc. [10]. Off-line processing of data, such as extracted ion chromatogram (EIC) data-mining method, plays a vital role in component identification [14,15]. These advantages have made LTQ-Orbitrap MS one of the most powerful approaches for the rapid identification and characterization of the multiple constituents found in TCMs [16]. It is well known that studies on flavonoids and alkaloids in *T. sinensis* by LC-MS have been widely reported, but little attention has been paid to its diterpenoids, mainly due to the complexity and variety of their skeletons, the diversity of their substituents, and a lack of corresponding reference standards. Hence, the characterization of diterpenoids in *T. sinensis* is of great significance.

In the present study, an HPLC-LTQ-Orbitrap data-acquisition approach combined with the EIC data-mining technique was established for comprehensive identification of the diterpenoids and their derivatives in *T. sinensis*.

## 2. Results and Discussion

Owing to the low content of diterpenoids in *T. sinensis*, a sensitive and reliable HPLC-LTQ-Orbitrap approach was established for determining their accurate masses and molecular formulas. In addition, the characteristic fragmentation pathways of diterpenoids were deduced from two obtained standards, and then used for the rapid identification and characterization of the other diterpenoids in *T. sinensis*. Furthermore, diterpenoid isomers were differentiated by an important parameter, Clog *P*, which is the absolute numerical value of the distribution coefficient of substances in a two-phase lipid-water system. Finally, target isolation of one diterpenoid glycoside named tinosineside A was performed based on the obtained results, which further confirmed the deduced fragmentation patterns. As a result, a total of 63 diterpenoids in *T. sinensis* were screened and divided into diterpenoid aglycones and diterpenoid glycosides (see supplementary materials and Table 1).

### 2.1. The Fragmentation Patterns of Reference Standards

Columbin and isocolumbin yielded their [M − H]^−^ ions at *m*/*z* 357.13326 (C_20_H_21_O_6_). Both of their deprotonated molecular ions generated a serial of fragment ions at *m*/*z* 342, *m*/*z* 339, *m*/*z* 313 and *m*/*z* 295, corresponding to [M − H − CH_3_]^−^, [M − H − H_2_O]^−^, [M − H − CO_2_]^−^ and [M − H − H_2_O − CO_2_]^−^. Columbin produced a fragment ion at *m*/*z* 329 through losing carbonyl, and the ion at *m*/*z* 329 loss of a CO_2_ produced its ESI-MS^2^ base peak ion at *m*/*z* 285. The ion at *m*/*z* 245 [M − H − 112]^−^ resulted from a Retro-Diels-Alder (RDA) cleavage fragmentation at the 1,4-position of the A-ring. Moreover, the product ion at *m*/*z* 245 generated the minor ion at *m*/*z* 217 by loss of one molecule of CO.

**Figure 1:**
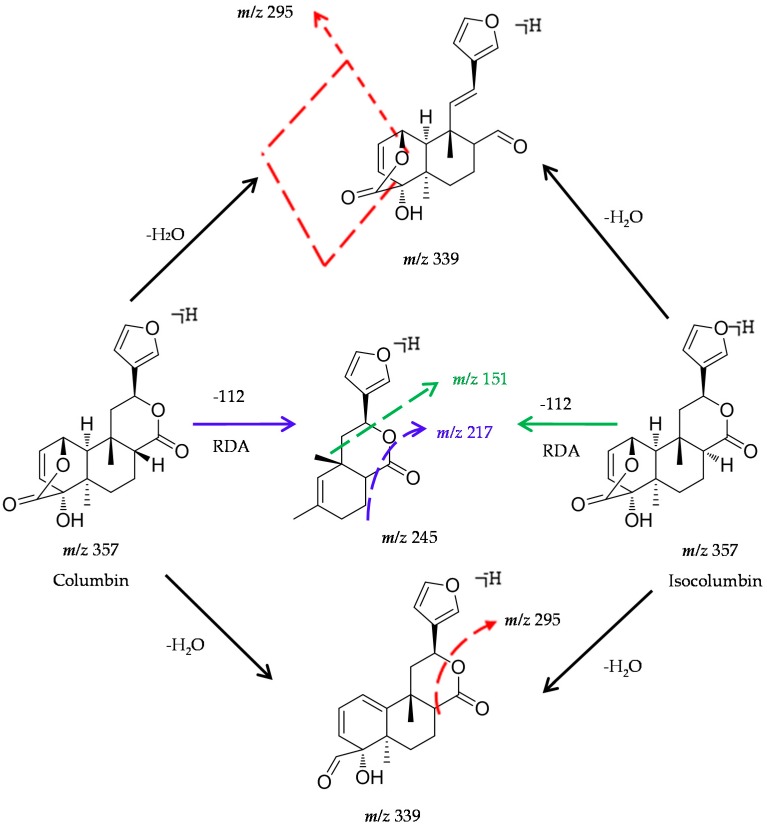
Proposed MS/MS mechanistic pathways for columbin and isocolumbin.

Meanwhile, isocolumbin produced its ESI-MS^2^ base peak ion at *m*/*z* 339 by neutral loss of H_2_O. After a RDA cleavage at A-ring and successive fracture of C_9–11_ (C_6_H_6_O, which was D-ring plus –C_2_H_2_), the prominent ion at *m*/*z* 151 was generated (Figure 1). Therefore, the characteristic fragment ions of reference standards were deduced, such as [M − H − 44]^−^ generated by loss of CO_2_ from lactone ring, as well as [M − H − 15]^−^, [M − H − 18]^−^, [M − H − 44 − 18]^−^, [M − H − 112]^−^, etc., According to these characteristic fragmentation patterns, diterpenoids in *T. sinensis* could be rapidly screened and identified.

### 2.2. Identification of Diterpenoids Aglycones

**Figure 2:**
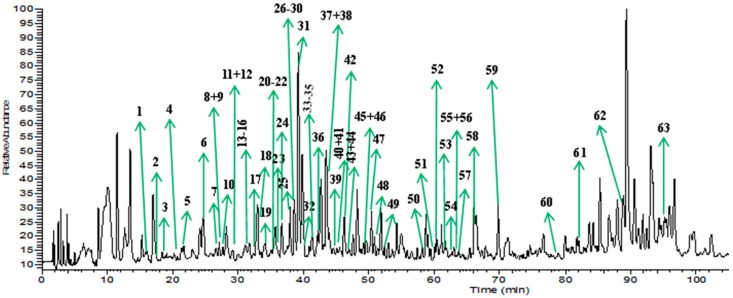
TIC chromatogram of *T. sinensis* in negative ion mode.

The molecular formulas of diterpenoids were predicted by a HRMS database built in-house. Combined with multi-stage mass spectrometry fragmentation information and bibliographic data, two categories of diterpenoids and its derivatives were screened and determined, including 10 diterpenoid aglycones and 53 diterpenoid glycosides. The typical total ion chromatogram (TIC) of *T. sinensis* in negative ion mode is presented in Figure 2.

Compounds **29** and **51** generated their [M − H]^−^ ions at *m*/*z* 357.13326 (C_20_H_21_O_6_). They were unambiguously assigned as isocolumbin and columbin, respectively, by comparison of their retention times, literature data and MS fragmentation data with their standards [17,18]. Isocolumbin and columbin constitute a pair of diastereomers, whose retention time and polarity are different under the present chromatographic conditions.

Compound **32** produced its [M − H]^−^ ion at *m*/*z* 417.15439 (C_22_H_25_O_8_). In CID mode, it further generated [M − H − CH_3_]^−^, [M − H − H_2_O − CO]^−^ and [M − H − H_2_O − CO − CH_3_]^−^ ion at *m*/*z* 402, *m*/*z* 371 and *m*/*z* 356, respectively. The ESI-MS^2^ base peak ion at *m*/*z* 181 (C_11_H_17_O_2_) was generated by RDA cleavage fragmentation from 1,4-position of the A-ring and fracture of C_9–11_ (C_6_H_4_O_3_). The product ion at *m*/*z* 181 loss of a methyl generated the prominent ion at *m*/*z* 166. Therefore, compound **32** was tentatively deduced as tinocapilactone B [19].

Compounds **40** and **41** produced their [M − H]^−^ ions at *m*/*z* 373.12817 (C_20_H_21_O_7_). Both of their deprotonated molecular ions generated a series of fragment ions at *m*/*z* 358 [M − H − CH_3_]^−^, *m*/*z* 343 [M − H − CO − H_2_]^−^, *m*/*z* 325 [M − H − 48 amu]^−^ and *m*/*z* 313 [M − H − 60 amu]^−^, respectively. In addition, compound **41** generated two minor ions at *m*/*z* 305 and *m*/*z* 261. By comparison with the MS fragmentation patterns obtained from the reference standards, compounds **40** and **41** were plausibly characterized as 8-hydroxycolumbin and 6-hydroxycolumbin, respectively.

Compound **57** showed the [M − H]^−^ ion at *m*/*z* 329.13835 (C_19_H_21_O_5_). Its ESI-MS^2^ base peak ion at *m*/*z* 314 was generated by loss of a methyl. The major fragment ions at *m*/*z* 311 and *m*/*z* 285 were yielded by neutral loss of H_2_O and CO_2_, respectively. Moreover, the product ion at *m*/*z* 285 generated the predominant product ion at *m*/*z* 191 due to the overall fracture of C_9–11_ (C_6_H_6_O). According to the literature data, it was tentatively identified as tinosponone [20].

Compounds **60** and **61** produced their respective [M − H]^−^ ion at *m*/*z* 329.17473 (C_20_H_25_O_4_) and *m*/*z* 347.18530 (C_20_H_27_O_5_). Both of their deprotonated molecular ions generated [M − H − H_2_O]^−^, [M − H − CO_2_]^−^ and [M − H − H_2_O − CO_2_]^−^ ions at *m*/*z* 311, *m*/*z* 329, *m*/*z* 285, *m*/*z* 303 and *m*/*z* 267, *m*/*z* 285, respectively. By comparing with the literature data, compounds **60** and **61** were tentatively identified as tinotufolin D and (2aβ,3*α*,5aβ,6β,7*α*,8a*α*)-6-2-(3-furanyl)ethyl-2a,3,4,5,5a,6,7,8,8a,8b- decahydro-2a,3-dihydroxy-6,7,8b-trimethyl-2*H*-naphtho-1,8-bcfuran-2-one, respectively [21].

Compound **62** gave a [M − H]^−^ ion at *m*/*z* 331.19038 (C_20_H_27_O_4_). Its MS^2^ spectrum produced the fragment ion at *m*/*z* 313, which involves the loss of H_2_O. The molecular ion yielded a series of fragment ions at *m*/*z* 287 [M − H − CO_2_]^−^, *m*/*z* 283 [M − H − H_2_O − H_2_CO]^−^ and *m*/*z* 271 [M − H − CH_4_ − CO_2_]^−^, suggesting the presence of -COOCH_3_. The base peak ion at *m*/*z* 285 was generated by losing water and carbon monoxide. The [M − H − C_6_H_6_O]^−^ ion at *m*/*z* 237 was generated by the fracture of C_9-11_ (C_6_H_6_O). Moreover, by comparison with the fragmentation patterns obtained from the two reference standards, compound **62** was tentatively identified as a new compound.

Compound **63** generated a [M − H]^−^ ion at *m*/*z* 363.21660 (C_21_H_31_O_5_). It generated a serial of fragment ions at *m*/*z* 345 [M − H − H_2_O]^−^, *m*/*z* 334 [M − H − 29 amu]^−^, *m*/*z* 317 [M − H − H_2_O − CO]^−^ and *m*/*z* 295 [M − H − 2H_2_O − OCH_4_]^−^. By comparison with the literature data, compound **63** was tentatively identified as tinotufolin C [21].

### 2.3. Identification of Diterpenoids Glycosides

Compounds **1** and **24** produced their [M – H]^−^ ions at *m*/*z* 765.29642 (C_37_H_49_O_17_). After the CID cleavage, both of their further fragmentations resulted in a [M − H − CO_2_]^−^ ion at *m*/*z* 721 and a [M − H − H_2_O − CO]^−^ ion at *m*/*z* 719, which were consistent with the characteristic fragmentation pathways of diterpenoids. According to the fragmentation patterns and the values of Clog *P*, compounds **1** and **24** were plausibly characterized as (2,3,4,6)-2-(acetoxymethyl)-6-(((2,4,6,6,10,10)-2-(furan-3-yl)-9,10-dimethoxy-7-(methoxycarbonyl)-6,10-dimethyl-4-oxododecahydro-1*H*-benzo[f]isochromen-6-yl)-oxy)tetrahydro- 2*H*-pyran-3,4,5-triyl triacetate and rumphioside D, respectively.

Compounds **2**, **6** and **39** produced their [M − H]^−^ ions at *m*/*z* 537.19665 (C_26_H_33_O_12_). They all produced the [M − H − H_2_O]^−^ and [M − H − H_2_O − CO]^−^ ions at *m*/*z* 519 and *m*/*z* 491. In addition, compounds **2** and **39** also yielded the [M − H − 162]^−^ ions at *m*/*z* 375 due to the overall fracture of dehydrated glucose, which was the ESI-MS^2^ base peak of compound **2**. As well as we known, [M − H − 162]^−^ was the characteristic ion of glycosides. Furthermore, compounds **2**, **6** and **39** generated the ions at *m*/*z* 490 [M − 2H − H_2_O − CO]^−^, *m*/*z* 357 [M − H − Glc]^−^, *m*/*z* 327 [M − H − Glc − 2CH_3_]^−^, *m*/*z* 297 [M − 2H − CO − Glc − OCH_3_]^−^; *m*/*z* 492 [M − 2H − CO_2_]^−^, *m*/*z* 490 [M − 2H − H_2_O − CO]^−^, *m*/*z* 469 [M − 2H − H_2_O − CO − OCH_3_]^−^, *m*/*z* 297 [M − 2H − CO − Glc − OCH_3_]^−^; *m*/*z* 493 [M − H − CO_2_]^−^, *m*/*z* 357 [M − H − Glc]^−^, *m*/*z* 341 [M − H − O − Glc]^−^, *m*/*z* 329 [M − H − CO − Glc]^−^, *m*/*z* 297 [M − 2H − CO − Glc −OCH_3_]^−^, respectively. By comparison with the literature data, compounds **2**, **6** and **39** were tentatively assigned as cordioside, cordifoliside D and borapetoside A, respectively [22,23].

Compounds **3**, **8**, **14**, **20**, **25**, **34**, **43**, **47** and **49** were all observed to possess the same [M − H]^−^ ions at *m*/*z* 597.21778 (C_28_H_37_O_14_). Firstly, they were divided into two categories based on whether they produced the [M − H − 112]^−^ ion at *m*/*z* 486 by the RDA cleavage from A-ring. Compound **47** generated its ESI-MS^2^ base peak ion at *m*/*z* 433 by losing dehydrated glucose. In addition, it also yielded [M − H − 2H_2_O]^−^ ion at *m*/*z* 561, [M − H − H_2_O − CO]^−^ ion at *m*/*z* 551, [M − H − 112]^−^ ion at *m*/*z* 486, [M − H − dehydrated Glc]^−^ ion at *m*/*z* 435, and so on. As far as we knew, there were no related literatures reported. And thus, compound **47** was finally deduced to be a new compound. Compounds **3**, **8**, **14**, **20**, **25**, **34**, **43** and **49** yielded their [M − H − H_2_O − CO]^−^ ions at *m*/*z* 551, which was the ESI-MS^2^ base peak of compounds **20** and **34**, and it also a high intense characteristic fragment ion of compound **49**. Compared with the prominent ions of them, there was no difficulty to deduce that compounds **34** and **49** have two lactone rings, which were different to the other compounds. Furthermore, the fragment ions further validated the above deduction. Finally, according to their Clog *P* values, they were tentatively identified and differentiated. By comparison with the bibliography and MS fragmentation data, compounds **3**, **8**, **14**, **20**, **25**, **34**, **43** and **49** were tentatively identified (Table 1) [24,25].

Compounds **4** and **46** generated their [M − H]^−^ ions at *m*/*z* 683.25456 (C_32_H_43_O_16_). Both of their deprotonated molecular ions yielded [M − H − H_2_O]^−^ ion at *m*/*z* 665, [M − H − H_2_O − CO]^−^ ion at *m*/*z* 637, [M − H − C_4_H_4_O]^−^ ion at *m*/*z* 615, [M − H − dehydrated Glc]^−^ ion at *m*/*z* 520, respectively. Moreover, compound **4** also dissociated into fragment ions [M − H − CO2]^−^ at *m*/*z* 639, [M − H − Glc − dehydrated xyl]^−^ at *m*/*z* 370 and [M − H − Glc − dehydrated xyl − CO_2_ − H_2_O]^−^ at *m*/*z* 309. According to the literature, compounds **4** and **46** were plausibly characterized as (2*R*,5*R*,6*R*,8*S*,9*S*,10*S*,12*S*)-15,16-epoxy-2-hydroxy-6-*O*-{β-d-xylopyranosyl(1→6)–d-glucopyranosyl}-cleroda-3,13(16),14-trien-17,12-olid-18-oic acid methyl ester and (2*R*,5*R*,6*R*,8*S*,9*S*,10*S*,12*S*)-15,16-epoxy-2-hydroxy-6-*O*-{β-d-glucopyranosyl-(1→6)-*α*-d-xylopyran-osyl}-cleroda-3,13(16),14-trien-17,12-olid-18-oic acid methyl ester, respectively [26].

Compounds **5** and **18** produced their [M − H]^−^ ions at *m*/*z* 513.19665 (C_24_H_33_O_12_). The dominant characteristic fragment ions were presented at *m*/*z* 333 [M – H − 180]^−^ and *m*/*z* 351 [M − H − 162]^−^ as the ESI-MS^2^ base peak corresponding to the cleavage from glucopyranosyl. After the CID cleavage, both of their further fragmentation resulted in [M − H − H_2_O]^−^ at *m*/*z* 495 and [M − H − Glc − CO]^−^ at *m*/*z* 305. The product ion at *m*/*z* 305 of compound **18** further generated the predominant ion at *m*/*z* 287 by losing one molecular of water. By comparison with the literature data and MS fragmentation data, compounds **5** and **18** were tentatively identified as 1-deacetyltinosposide A and (2*S*,6*R*,6a*R*,7*R*,9*S*,10*R*,10a*R*,10b*S*)-2-(furan-3-yl)-6,9,10–trihydroxy-10b-methyl-7-(((2*R*,3*R*,4*S*,5*S*,6*R*)-3,4,5-trihydroxy-6-(hydroxymethyl)tetrahydro-2*H*-pyran-2-yl)oxy)decahydro-1*H*-benzo[*f*]isochromen-4(2*H*)-one, respectively [4].

Compounds **7**, **10**, **15** and **17** gave their [M − H]^−^ ions at *m*/*z* 567.20721 (C_27_H_35_O_13_). They all produced the [M − H − H_2_O − CO]^−^ and [M − H − Glc]^−^ ions at *m*/*z* 521 and *m*/*z* 405. Besides, compounds **7** and **10** generated *m*/*z* 552 by losing a methyl, and the ion at *m*/*z* 341 was generated by losing a series of fragment ions of glucose residue, 2H_2_O, and CO. Compounds **7** and **15** were also generated [M − H − Glc − CO]^−^ ions at *m*/*z* 359, which was the ESI-MS^2^ base peak of compound **7**. Meanwhile, compound **7** generated the minor ion at *m*/*z* 329. Compound **17** produced its ESI-MS^2^ base peak by losing two molecular of methyl, one molecular of water and carbonyl at *m*/*z* 491. Combined with bibliography data and fragmentation pathways, these four compounds were tentatively deduced as tinospinoside D, amritoside C, rumphioside A and rumphioside F, respectively [25].

Compounds **9**, **11**, **13**, **19**, **35** and **55** all produced their [M − H]^−^ ions at *m*/*z* 551.21230 (C_27_H_35_O_12_). Compounds **9**, **13** and **19** produced their ESI-MS^2^ base peak ions at *m*/*z* 389 [M − H − dehydrated Glc]^−^, and further generated a series of fragment ions by losing methyl, water, carbon dioxide units at the mean time. According to the literature and the values of Clog *P*, compounds **9**, **13** and **19** were plausibly characterized as borapetoside B, tinosposinenside A and (2*R*,5*R*,6*R*,8*R*,9*S*,10*S*,12*S*)-15,16-epoxy-2-hydroxy-6-*O*-(β-d-glucopyranosyl)-cleroda-3,13(16), 14-trien-17,12-olid-18-oic acid methyl ester, respectively [26]. Compounds **11** and **55** generated their ESI-MS^2^ base peak ions at *m*/*z* 505 by losing water and carbonyl, and their deprotonated molecular ions loss of glucose residue at *m*/*z* 389. According to the fragmentation pathways and the values of Clog *P*, compounds **11** and **55** were tentatively identified as tinospinoside B and tinospinoside C, respectively. According to the above methods, compound **35** was tentatively defined as rumphioside I.

Compounds **12** and **21** yielded their [M − H]^−^ ions at *m*/*z* 535.18100 (C_26_H_31_O_12_). Compound **12** produced its ESI-MS^2^ base peak ion at *m*/*z* 373 by loss of a dehydrated glucose and then generated two minor ions at *m*/*z* 345 [M − H − dehydrated Glc − CO]^−^ and *m*/*z* 313 [M − H − O − dehydrated Glc − CO_2_]^−^. While compound **21** generated a series of fragment ions at *m*/*z* 520 [M − H − CH_3_]^−^, *m*/*z* 517 [M − H − H_2_O]^−^, *m*/*z* 373 [M − H − dehydrated Glc]^−^ and *m*/*z* 329 [M − H − dehydrated Glc − CO_2_]^−^. According to the fragmentation patterns and the values of Clog *P*, compounds **12** and **21** were tentatively characterized as (2,4,6,6,7,7,8,9,9,9)-2-(furan-3-yl)-6a,9b-dimethyl-6-(((2,3,4,5,6)-3,4,5-trihydroxy-6-(hydroxymethyl)tetrahydro-2*H*-pyran-2-yl)oxy)decahydro-1*H*-9,7-(epoxymethano) oxireno[2′,3′:4,5]benzo[1,2-f]isochromene-4,11(2*H*)-dione and palmatoside F, respectively.

Compound **16** produced its [M − H]^−^ ion at *m*/*z* 521.20173 (C_26_H_33_O_11_). A high intense characteristic fragment ion was present at *m*/*z* 359 [M – H − 162]^−^ as its ESI-MS^2^ base peak corresponding to the cleavage of the dehydrated glucose. The product ion at *m*/*z* 359 successively generated the predominant product ions at *m*/*z* 344 and *m*/*z* 341 by losing a methyl and one molecular of water. According to the literature data, compound **16** was tentatively identified as furanoid diterpene glycoside [27].

Compound **22** generated its [M − H]^−^ ion at *m*/*z* 527.21230 (C_25_H_35_O_12_). Upon CID mode, its further fragmentation resulted in [M − H − H_2_O]^−^ ion at *m*/*z* 509, [M − H − H_2_O − CH_3_]^−^ ion at *m*/*z* 494, [M – H − H_2_O − CO]^−^ ion at *m*/*z* 481, [M − H − Glc]^−^ ion at *m*/*z* 347, and [M − H − Glc − CH_2_O − CO − CH_4_]^−^ ion at *m*/*z* 273. As far as we knew, there were no related literatures reported. Thus, compound **22** was tentatively deduced to be a new compound.

Compounds **23**, **31** and **37** yielded their [M − H]^−^ ions at *m*/*z* 555.20721 (C_26_H_35_O_13_). Compound **31** produced its ESI-MS^2^ base peak ion at *m*/*z* 513 [M − Ac]^−^, and the *m*/*z* 513 ion generated its predominant ions at *m*/*z* 495 [M − H − CH_2_CO − H_2_O]^−^ and *m*/*z* 333 [M − H − CH_2_CO − dehydrated Glc]^−^ due to the overall fracture of a hydroxy and glycoside bond. The ion at *m*/*z* 333 generated two minor ions at *m*/*z* 315 and *m*/*z* 305 through losing H_2_O and CO, respectively. Besides, its [M − H]^−^ ion also yielded characteristic fragment ion at *m*/*z* 393 [M − H − 162]^−^ by losing a dehydrated glucose. By comparison with the literature data, compound **31** was tentatively identified as tinosineside A [24]. Compounds **23** and **37** produced their fragment ions at *m*/*z* 537 [M − H − H_2_O]^−^, *m*/*z* 513 [M − CH_3_ − CO]^−^, *m*/*z* 495 [M − H − CH_3_ − COOH]^−^ and *m*/*z* 393 [M − H − dehydrated Glc]^−^. Furthermore, compound **23** generated the fragment ions at *m*/*z* 375 [M − H–H_2_O–Glc]^−^, *m*/*z* 305 [M − H–dehydrated Glc–CH_4_ − CO − CO_2_]^−^ and *m*/*z* 297 [M − H − H_2_O − Glc − CO_2_ − CH_4_]^−^ or [M − H − 2H_2_O − Glc − CH_2_CO]^−^. According to the fragment ions and the values of Clog *P*, compounds **23** and **37** were tentatively identified as amritoside A and (1,2,7,8)-1-(2-(furan-3-yl)-2-hydroxyethyl)-2-hydroxy-5-(methoxycarbonyl)-1-methyl-7-(((3,4,6)-3,4,5-trihydroxy-6-(hydroxymethyl)tetrahydro-2*H*-pyran-2-yl)oxy)-1,2,3,4,6,7,8,8-octahydronaphthalene-2-carboxylic acid, respectively.

Compound **26** produced its [M − H]^−^ ion at *m*/*z* 523.21738 (C_26_H_35_O_11_). Its ESI-MS^2^ base peak ion at *m*/*z* 361 was yielded by loss of a dehydrated glucose. In addition, it generated a series fragment ions, such as *m*/*z* 508, *m*/*z* 505, *m*/*z* 347, *m*/*z* 343 and *m*/*z* 329, involving the loss of a methyl group, a water molecule, and glucose. By comparing with the fragmentation patterns obtained from two reference standards, compound **26** was tentatively identified as sagittatayunnanoside D. Similarly, based on the literature data, compound **27** was tentatively identified as borapetoside H [28].

Compounds **28** and **33** exhibited their [M − H]^−^ ions at *m*/*z* 549.19665 (C_27_H_33_O_12_). Both of their deprotonated molecular ions generated a serial of fragment ions at *m*/*z* 531 [M − H − H_2_O]^−^, *m*/*z* 503 [M − H − H_2_O − CO]^−^, *m*/*z* 481 [M − H − 2H_2_O − CH_3_OH]^−^ and *m*/*z* 387 [M − H − dehydrated Glc]^−^, respectively. Meanwhile, a product ion at *m*/*z* 513 [M − H − 2H_2_O]^−^ was observed in the ESI-MS/MS spectra of compound **33**, suggesting there was two hydroxy groups at the skeleton. According to the literature data and the values of Clog *P*, compounds **28** and **33** were tentatively identified as (5*R*,6*R*,8*S*,9*R*,10*R*,12*S*)-15,16-epoxy-2-oxo-6-*O*-(β-d-glucopyranosyl)–cleroda-3,13(16),14-trien-17,12 -olid-18-oic acid methyl ester and tinoscorside C, respectively [26].

Compound **30** generated its [M − H]^−^ ion at *m*/*z* 519.18608 (C_26_H_31_O_11_). The dominant characteristic fragment ion was present at *m*/*z* 357 [M − H − 162]^−^ as its ESI-MS^2^ base peak, corresponding to the cleavage from the dehydrated glucose. Meanwhile, the product ions [M − H − H_2_O]^−^ (lose of water) at *m*/*z* 501 and [M − H − 46]^−^ (lose of water and carbon monoxide) at *m*/*z* 473 were monitored, which were in accordance with the diterpenoids glycosides cracking patterns, suggesting this compound might be tinoside.

Compounds **36** and **44** yielded their [M − H]^−^ ions at *m*/*z* 535.21738 (C_27_H_35_O_11_). Both of their deprotonated molecular ions generated a serial of fragment ions at *m*/*z* 520 [M − H − CH_3_]^−^, *m*/*z* 488 [M − 2H − CO − H_2_O]^−^, *m*/*z* 373 [M − H − dehydrated Glc]^−^, *m*/*z* 359 [M − H − dehydrated Glc − CH_2_]^−^, *m*/*z* 358 [M − H − dehydrated Glc − CH_3_]^−^, *m*/*z* 355 [M − H − Glc]^−^ and *m*/*z* 341 [M − H − Glc − CH_2_]^−^. According to the literature data and the values of Clog *P*, compounds **36** and **44** were tentatively identified as borapetoside C and tinocrisposide [29].

Compound **38** gave a [M − H]^−^ ion at *m*/*z* 581.22286 (C_28_H_37_O_13_). Its major ions in the MS^2^ spectrum were *m*/*z* 563 [M − H − H_2_O]^−^, *m*/*z* 535 [M − H − H_2_O − CO]^−^, *m*/*z* 419 [M − H − dehydrated Glc]^−^, *m*/*z* 373 [M − H − Glc − CO]^−^ and *m*/*z* 343 [M − Glc − CH_2_CO_2_]^−^, suggesting this compound might contain the glucose and acetyl fragments. Comparison with the parent nucleus and fragmentation patterns, compound **38** was tentatively identified as tinosposinenside B.

Compound **42** exhibited its [M − H]^−^ ion at *m*/*z* 537.23303 (C_27_H_37_O_11_). Its ESI-MS^2^ base peak ion at *m*/*z* 375 was produced by losing dehydrated glucose, and the product ion at *m*/*z* 375 further generated two minor ions at *m*/*z* 360 and *m*/*z* 357 by losing CH_3_ and H2O, respectively. According to the fragmentation pathways, compound **42** was tentatively identified as boropetoside G. Likewise, compound **48** was tentatively defined as tinosposinenside C.

Compound **45** produced its [M − H]^−^ ion at *m*/*z* 597.20002 (C_28_H_37_O_12_S), which exhibited the characteristic fragment ion [M − H − C_8_H_14_O_6_S]^−^ at *m*/*z* 359 suggesting the overall fracture of side chain. S-diterpenoids glycosides were very rare in *Tinospora*, and there only two compounds were reported in this family so far [30]. By comparison with the fragmentation pathways, compound **45** was plausibly described as cordifolide A.

Compounds **50** and **59** produced their [M − H]^−^ ions at *m*/*z* 623.23343 (C_30_H_39_O_14_). According to the respective fragmentation pathways and the values of Clog *P*, compounds **50** and **59** were tentatively identified as 6′-*O*-lactoylborapetoside B and 2-*O*-lactoylborapetoside B, respectively.

Compounds **52**–**54** produced their respective [M − H]^−^ ion at *m*/*z* 517.17043 (C_26_H_29_O_11_), *m*/*z* 491.19117 (C_25_H_31_O_10_) and *m*/*z* 715.28077 (C_33_H_47_O_17_). None but compound **53** exhibited the [M − H + HCOOH]^−^ adduct ion at *m*/*z* 537.19665 (C_26_H_33_O_12_). Take compound **52** for example, its major ions in ESI-MS^2^ spectrum were *m*/*z* 499 [M − H − H_2_O]^−^, *m*/*z* 473 [M − H − CO_2_]^−^, *m*/*z* 471 [M − H − H_2_O − CO]^−^, *m*/*z* 455 [M − H − CO_2_ − H_2_O]^−^, *m*/*z* 355 [M − H − dehydrated Glc]^−^, *m*/*z* 162 −C_6_H_10_O_5_ and *m*/*z* 151 –C_5_H_11_O_5_, suggesting this compound might contain glucose and undergo a RDA coverage from the A-ring at the 1,4-position. According to the above analysis, compounds **52**–**54** were tentatively defined as tinospinoside E, tinosporaside and sagittatayunnanoside B, respectively.

Compounds **56** and **58** generated their respective [M − H]^−^ ion at *m*/*z* 671.29094 (C_32_H_47_O_15_) and 509.23812 (C_26_H_37_O_10_). The defference between of these molecules was 162 Da, suggesting that compound **56** had one more glucose unit than compound **58**. Both of them generated the same ESI-MS^2^ base peak ions at *m*/*z* 329 due to the overall fracture of glycoside bond. Furthermore, the product ion at *m*/*z* 329 generated a predominant ion at *m*/*z* 301 by splitting off CO. Comparing with the parent nucleus and respective fragmentation pathways, compounds **56** and **58** were plausibly identified as sagittatayunnanoside C and sagittatayunnanoside A, respectively.

### 2.4. Target Isolation and Further Verification of Diterpenoids Fragmentation Patterns

**Figure 3:**
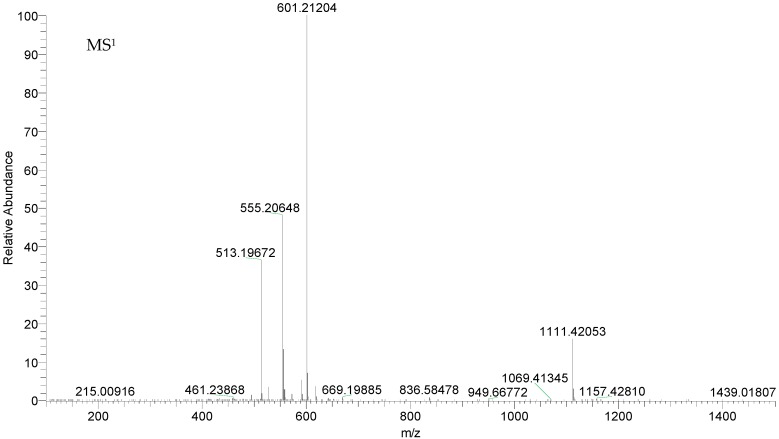

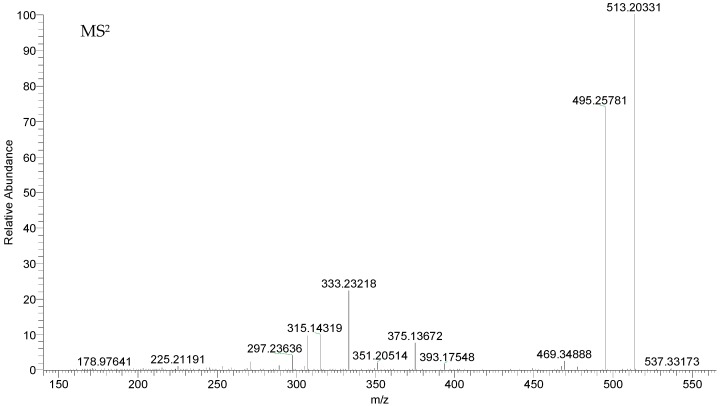
Spectra of ion fragments in MS^n^ analysis of tinosineside A in negative ion mode.

Tinosineside A generated its deprotonated molecular ion [M − H]^−^ at *m*/*z* 555.20721 (C_26_H_35_O_13_, mass error within 3 ppm). It firstly produced the ESI-MS^2^ base peak ion at *m*/*z* 513 [M − Ac]^−^ and further generated the ions at *m*/*z* 495 and *m*/*z* 375 by losing one molecule of water and dehydrated glucose. The ion at *m*/*z* 351 generated minor ions at *m*/*z* 333, *m*/*z* 315 and *m*/*z* 307 through losing H_2_O, 2H_2_O and CO2, respectively. Besides, its [M − H]^−^ ion also yielded characteristic fragment ion at *m*/*z* 393 [M − H − 162]^−^ by losing a dehydrated glucose. The fragmentation pathway was consistent with deduced of compound 31, which further proved the validity of the results (Figure 3).

## 3. Materials and Methods

### 3.1. Chemicals

The reference standards of columbin and isocolumbin (purity over 98%) were procured from Tauto Biotech (Shanghai, China).The structures of these standards were presented in Figure 1. HPLC grade formic acid, acetonitrile and methanol were purchased from Fisher Scientific (Fair Lawn, NJ, USA). Ultrapure water was purchased from Hangzhou Wahaha Group Co., Ltd. (Hangzhou, Zhejiang, China). Material of *T. sinensis* was purchased from Anguo Linshi Medicinal Materials Co., Ltd. (Anguo, China) and then authenticated by Professor Chun-sheng Liu in Beijing University of Chinese Medicine.

### 3.2. Sample Preparation

#### 3.2.1. Standard Solutions

The standard solutions of columbin and isocolumbin were prepared in methanol at appropriate concentrations.

#### 3.2.2. Sample Solutions

Powdered dried alcoholic extracts of *T. sinensis* were weighed accurately (0.13 g) and refluxed with a tenfold excess of ethanol/water (70:30, *v*/*v*) three times, and placed in 20 mL of methanol/water (70:30, *v*/*v*). The mixture was then extracted in an ultrasonic bath at room temperature for 0.5 h, and the same solvent was added to compensate for the weight lost during the extraction. The extract was filtered and evaporated to near dryness, then placed on a C_18_ Solid Phase Extraction (SPE) column (J.T.Baker, Phillipsburg, NJ, USA), which was washed with 4 mL distilled water and 4 mL methanol. The methanol eluent was filtered through a 0.22 µm membrane for analysis. All of the solutions were stored at 4 °C and brought to room temperature before analysis.

### 3.3. Instrumentation and Condition

HPLC analysis was carried out on a DIONEX Ultimate 3000 UHPLC system (Thermo Fisher Scientific, Waltham, MA, USA) with a binary pump and an autosampler. The samples were separated on a Sunfire C18 column (250 mm × 4.6 mm i.d., 5 μm, Waters Corporation, Milford, MA, USA) at room temperature. The mobile phase consisted of acetonitrile (**B**) and 0.1% (*v*/*v*) formic acid in water (**A**) with the elution gradient set as follows: 0–5 min, 8–12% **B**; 5–25 min, 12–16% **B**, 25–45 min, 16–25% **B**; 45–75 min, 25–46% **B**; 75–80 min, 46–58% **B**; 80–95 min, 58–65% **B**; 95–105 min. The flow rate was set as 1.0 mL/min.

A LTQ-Orbitrap XL mass spectrometer (Thermo Scientific, Bremen, Germany) was connected to the HPLC system via an electrospray ionization (ESI) interface. The effluent was introduced into the ESI source in a post-column splitting ratio of 1:4. Full scan data acquisition was performed from *m*/*z* 100 to 1500 in negative ion mode. The important ESI parameters were set as follows: capillary temperature, 350 °C; sheath gas (nitrogen) flow, 30 arb.; auxiliary gas (nitrogen) flow, 10 arb.; electrospray voltage, 3.0 kV; capillary voltage, −35 V; tube lens voltage, −110 V. The resolution of Orbitrap analyzer was set at 30,000 with data-dependent ESI-MS^2^ analysis triggered by the three most abundant ions from one-stage mass spectrometry scanning. Collision-induced dissociation (CID) was performed in LTQ with an activation *q* of 0.25 and activation time of 30 ms. The isolation width was 2 amu, and the normalized collision energy was set to 35%.

### 3.4. Peak Selections and Data Processing

A Thermo Xcalibur 2.1 workstation was used for the data acquisition and processing, In order to obtain as many fragment ions of the diterpenoids as possible, the peaks detected with intensity over 30,000 were selected for identification. The chemical formulas for all parent ions of the selected peaks were calculated from the accurate mass using a formula predictor by setting the parameters as follows: C (0–50), H (0–100), O (0–30), S (0–2), N (0–2). Other elements were not considered because they are rarely present in diterpenoids. Furthermore, diterpenoids isomers were differentiated by an important Clog *P* parameter obtained from Chemdraw to distinguish their polarity. And the exact mass error of all determined compounds was within 3 ppm.

### 3.5. Extraction and Isolation of Tinosineside A

The air-dried stems (10.0 kg) were extracted three times with tenfold excess of 70% EtOH under reflux for 2 h each at 80 °C. The combined extract was evaporated under reduced pressure to obtain a crude residue. This residue was further dispersed in H_2_O, and then successively extracted with CHCl_3_, EtOAc, *n*-BuOH and MeOH. The *n*-BuOH extract was passed through an AB-8 macroporous resin column and then washed with H_2_O, 30% EtOH, 50% EtOH, 70% EtOH and 95% EtOH. The 30% EtOH fraction was further purified by silica gel column chromatography with elution by CHCl_3_–MeOH (15:1→4:1, *v*/*v*) to give the diterpenoid glycoside named tinosineside A as a white powder.

## 4. Conclusions

In this study, a sensitive HPLC-LTQ-Orbitrap coupled with EIC data-mining method was established for the rapid characterization of diterpenoids in *T. sinensis*. According to the characteristic fragmentation pathways of two reference standards, all diterpenoids were divided into two categories, namely diterpenoid aglycones and diterpenoid glycosides. Furthermore, an important parameter Clog *P* was used to differentiate the isomers of diterpenoids. As a result, 63 diterpenoids were preliminarily identified, including 48 known compounds and 15 new compounds. These compounds included 10 diterpenoid aglycones, 53 diterpenoid glycosides. Finally, target isolation of one diterpenoid glycoside named tinosineside A was performed based on the obtained results, which further confirmed the deduced fragmentation patterns and identified profile of diterpenoids in *T. sinensis*. This represents the first systematic report of diterpenoids in *T. sinensis*. The results indicated that the established method could be employed as a rapid, effective technique to screen and identify diterpenoids in *T. sinensis*. The study also provided significant guidance for the analysis of other herbal medicines or preparations.

## Figures and Tables

**Table 1:** Summary of chemical constituents identified in *Tinospora sinensis* by HPLC-LTQ-Orbitrap.

NO.	t_R_/min	Identification	Empirical Formula	Proposed Ions	Experimental Mass *m*/z	Theoretical Mass *m*/*z*	Mass Error (×10^−6^)	MS^2^ Data (Measured)
**1**	16.13	(2,3,4,6)-2-(Acetoxymethyl)-6-(((2,4,6,6,10,10)-2-(furan-3-yl)-9,10-dimethoxy-7-(methoxycarbonyl)-6,10-dimethyl-4-oxododecahydro-1*H*-benzo[f]isochromen-6-yl)oxy)-tetrahydro-2*H*-pyran-3,4,5-triyl triacetate *	C_37_H_49_O_17_	[M − H]^−^	765.29578	765.29642	0.836	747(10), 737(48), 721(38), 720(43), 719(100), 697(5), 672(11), 555(18)
**2**	17.69	Cordioside	C_26_H_3__3_O_12_	[M − H]^−^	537.19592	537.19665	1.359	519(4), 491(8), 490(13), 375(100), 357(43), 327(88), 297(4)
**3**	18.55	(4,6,6,7,9,10,10,10)-2-(Furan-3-yl)-7-hydroxy-10b-methyl-4-oxo-6-(((3,5,6)-3,4,5-trihydroxy-6-(hydroxymethyl)-tetrahydro-2*H*-pyran-2-yl)-oxy)dodecahydro-1*H*-benzo-[f]isochromene-9,10-diyl diacetate *	C_28_H_3__7_O_14_	[M − H]^−^	597.21661	597.21778	1.959	579(3), 551(60), 476(7), 435(10), 389(96), 359(100)
**4**	20.51	(2,5,6,8,9,10,12)-15,16-Epoxy-2-hydroxy-6-*O*-{β-d-xylopyranosyl(1→6)-d-glucopyranosyl}-cleroda-3,13(16),14-trien-17,12-olid-18-oic acid methyl ester *	C_32_H_4__3_O_16_	[M − H]^−^	683.25372	683.25456	1.229	665(68), 639(12), 637(16), 615(95), 520(100), 519(79), 370(15), 309(37)
**5**	21.89	1-Deacetyltinosposide A	C_24_H_3__3_O_12_	[M − H]^−^	513.19678	513.19665	−0.253	495(3), 351(12), 333(100), 307(11), 305(5), 271(5)
**6**	25.01	Cordifoliside D	C_26_H_3__3_O_12_	[M − H]^−^	537.19623	537.19665	0.782	519(38), 492(22), 491(100), 490(71), 469(71), 297(38)
**7**	26.40	Tinospinoside D	C_27_H_3__5_O_13_	[M − H]^−^	567.20660	567.20721	1.075	552(1), 521(9), 404(0.5), 359(100), 341(2), 329(2)
**8**	26.55	(6,6,10,10,10)-Methyl 10-acetoxy-2-(furan-3-yl)-9-hydroxy-10b-methyl-4-oxo-6-(((3,5,6)-3,4,5-trihydroxy-6-(hydroxymethyl)tetrahydro-2*H*-pyran-2-yl)oxy)-dodecahydro-1*H*-benzo[f]-isochromene-7-carboxylate *	C_28_H_3__7_O_14_	[M − H]^−^	597.21741	597.21778	0.619	565(4), 551(21), 550(25), 535(10), 463(12), 435(12), 389(100), 388(12)
**9**	26.98	Borapetoside B	C_27_H_3__5_O_12_	[M − H]^−^	551.21204	551.21230	0.471	536(5), 533(2), 507(3), 389(100), 388(15), 371(2), 370(7), 329(2)
**10**	27.65	Amritoside C	C_27_H_3__5_O_13_	[M − H]^−^	567.20612	567.20721	1.921	552(6), 529(20), 521(100), 404(10), 341(4)
**11**	29.43	Tinospinoside B	C_27_H_3__5_O_12_	[M − H]^−^	551.21298	551.21230	−1.233	533(9), 532(31), 515(12), 505(100), 389(8), 344(8)
**12**	29.52	(2,4,6,6,7,7,8,9,9,9)-2-(furan-3-yl)-6a,9b-dimethyl-6-(((2,3,4,5,6)-3,4,5-trihydroxy-6-(hydroxymethyl)-tetrahydro-2*H*-pyran-2-yl)oxy)decahydro-1*H*-9,7-(epoxymethano)-oxireno[2′,3′:4,5]benzo[1,2-f]isochromene-4,11(2*H*)-dione *	C_26_H_3__1_O_12_	[M − H]^−^	535.18036	535.18100	1.196	517(0.5), 489(2), 467(2), 373(100), 345(1), 343(2), 313(2)
**13**	30.82	Tinosposinenside A	C_27_H_3__5_O_12_	[M − H]^−^	551.21173	551.21230	1.034	536(5), 533(3), 507(3), 389(100), 374(11), 371(16), 359(2), 341(7)
**14**	31.03	(6,6,7,9,10,10,10)-2-(Furan-3-yl)-7-hydroxy-10b-methyl-4-oxo-6-(((3,5,6)-3,4,5-trihydroxy-6-(hydroxymethyl)-tetrahydro-2*H*-pyran-2-yl)oxy)dodecahydro-1*H*-benzo-[f]isochromene-9,10-diyl diacetate *	C_28_H_3__7_O_14_	[M − H]^−^	597.21692	597.21778	1.440	579(4), 561(11), 551(13), 529(19), 515(5), 477(6), 435(12), 389(82), 359(100)
**15**	31.41	Rumphioside A	C_27_H_3__5_O_13_	[M − H]^−^	567.20685	567.20721	0.635	549(14), 548(25), 535(25), 521(34), 405(46), 404(100), 359(56)
**16**	31.97	Furanoid diterpene glycoside	C_26_H_3__3_O_11_	[M − H]^−^	521.20099	521.20173	1.420	506(1), 359(100), 345(3), 344(4), 341(3)
**17**	32.03	Rumphioside F	C_27_H_3__5_O_13_	[M − H]^−^	567.20642	567.20721	1.393	549(22), 521(47), 491(100), 405(11), 404(18), 387(19)
**18**	33.06	(2,6,6,7,9,10,10,10)-2-(Furan-3-yl)-6,9,10-trihydroxy-10b-methyl-7-(((2,3,4,5,6)-3,4,5-trihydroxy-6-(hydroxyl-methyl)tetrahydro-2*H*-pyran-2-yl)oxy)decahydro-1*H*-benzo[*f*]isochromen-4(2*H*)-one *	C_24_H_3__3_O_12_	[M − H]^−^	513.19635	513.19665	0.585	495(3), 467(1), 351(100), 305(60), 287(7), 161(3)
**19**	34.12	(2*R*,5*R*,6*R*,8*R*,9*S*,10*S*,12*S*)-15,16-Epoxy-2-hydroxy-6-*O*-(β-d-glucopyranosyl)-cleroda-3,13(16),14-trien-17,12-olid-18-oic acid methyl ester	C_27_H_3__5_O_12_	[M − H]^−^	551.21185	551.21230	0.816	533(2), 519(3), 505(4), 482(4), 389(100), 327(67)
**20**	35.07	Tinosineside B	C_28_H_3__7_O_14_	[M − H]^−^	597.21716	597.21778	1.038	578(20), 551(100), 529(10), 517(12), 461(25), 179(49)
**21**	35.26	Palmatoside F	C_26_H_3__1_O_12_	[M − H]^−^	535.17987	535.18100	2.111	520(4), 517(2), 488(3), 373(93), 329(100)
**22**	35.35	(2,6,7,9,10,10,10)-2-(Furan-3-yl)-7,10-dihydroxy-9-methoxy-10b-methyl-6-(((2,3,4,5,6)-3,4,5-trihydroxy-6-(hydroxymethyl)tetrahydro-2*H*-pyran-2-yl)oxy)deca-hydro-1*H*-benzo[*f*]isochromen-4(2*H*)-one *	C_25_H_3__5_O_12_	[M − H]^−^	527.21241	527.21230	−0.209	511(14), 509(3), 494(14), 481(100), 347(6), 300(7)
**23**	35.80	Amritoside A	C_26_H_3__5_O_13_	[M − H]^−^	555.20624	555.20721	1.747	537(5), 513(34), 495(53), 467(19), 393(100), 375(5), 305(74), 287(7)
**24**	36.87	Rumphioside D	C_37_H_49_O_17_	[M − H]^−^	765.29431	765.29642	2.757	747(19), 734(20), 721(37), 719(41), 718(100), 697(18), 600(26), 418(4), 393(11)
**25**	38.12	(4,6,6,9,10,10)-Methyl 9-acetoxy-2-(furan-3-yl)-4a- hydroxy-10b-methyl-4-oxo-6-(((3,5,6)-3,4,5-trihydroxy-6-(hydroxymethyl)tetrahydro-2*H*-pyran-2-yl)oxy)-dodecahydro-1*H*-benzo[f]isochromene-7-carboxylate *	C_28_H_3__7_O_14_	[M − H]^−^	597.21619	597.21778	2.662	582(73), 579(9), 556(41), 551(28), 550(72), 434(31), 416(33), 389(75), 329(100)
**26**	38.15	Sagittatayunnanoside D	C_26_H_3__5_O_11_	[M − H]^−^	523.21667	523.21738	1.357	508(1), 505(1), 361(100), 347(8), 343(4), 329(4)
**27**	38.26	Borapetoside H	C_33_H_4__5_O_17_	[M − H]^−^	713.26588	713.26512	−1.066	694(12), 668(18), 667(29), 666(100), 645(23), 551(21), 533(6), 389(6)
**28**	38.38	(5*R*,6*R*,8*S*,9*R*,10*R*,12*S*)-15,16-Epoxy-2-oxo-6-*O*-(β-d-glucopyranosyl)–cleroda-3,13(16) 14-trien-17,12-olid-18-oic acid methyl ester	C_27_H_3__3_O_12_	[M − H]^−^	549.19592	549.19665	1.329	531(0.4), 503(5), 481(3), 387(100), 249(1)
**29**	38.80	Isocolumbin ^a^	C_20_H_2__1_O_6_	[M − H]^−^	357.13321	357.13326	0.140	342(9), 339(2), 313(1), 311(13), 151(100), 135(37)
**30**	38.84	Tinoside	C_26_H_3__1_O_11_	[M − H]^−^	519.18542	519.18608	1.271	501(0.1), 473(0.3), 358(0.4), 357(100)
**31**	39.44	Tinosineside A	C_26_H_3__5_O_13_	[M − H]^−^	555.20581	555.20721	2.522	513(100), 495(69), 393(2), 375(8), 333(28), 315(10), 307(13), 305(1)
**32**	40.03	Tinocapilactone B	C_22_H_2__5_O_8_	[M − H]^−^	417.15411	417.15439	0.671	402(42), 371(9), 356(2), 181(100), 166(34), 151(16)
**33**	40.61	Tinoscorside C	C_27_H_3__3_O_12_	[M − H]^−^	549.19586	549.19665	1.438	531(7), 513(2), 503(13), 481(10), 417(5), 387(100)
**34**	40.79	(1,3,10,10)-9-(2-Methoxy-2,5-dihydrofuran-3-yl)-10a-methyl-4,7-dioxo-3-(((2,3,4,5,6)-3,4,5-trihydroxy-6-(hydroxymethyl)tetrahydro-2*H*-pyran-2-yl)oxy)tetra-decahydroisobenzofuro[7,1-fg]isochromen-1-yl acetate *	C_28_H_3__7_O_14_	[M − H]^−^	597.21655	597.21778	2.060	579(51), 551(100), 528(39), 471(12)
**35**	41.60	Rumphioside I	C_27_H_3__5_O_12_	[M − H]^−^	551.21210	551.21230	0.363	533(9), 519(11), 505(2), 482(3), 339(100), 324(5)
**36**	42.17	Borapetoside C	C_27_H_3__5_O_11_	[M − H]^−^	535.21637	535.21738	1.887	520(1), 517(4), 488(1), 373(100), 359(5), 358(7), 355(2), 341(5)
**37**	43.22	(1,2,7,8)-1-(2-Ffuran-3-yl)-2-hydroxyethyl)-2-hydroxy-5-(methoxycarbonyl)-1-methyl-7-(((3,4,6)-3,4,5-trihydroxy-6-(hydroxymethyl)tetrahydro-2*H*-pyran-2-yl)oxy)-1,2,3,4,6,7,8,8-octahydronaphthalene-2-carboxylic acid *	C_26_H_3__5_O_13_	[M − H]^−^	555.20630	555.20721	1.639	537(0.2), 513(1), 495(100), 393(0.2), 333(1), 297(2), 178(0.2)
**38**	43.99	Tinosposinenside B	C_28_H_3__7_O_13_	[M − H]^−^	581.22162	581.22286	2.133	563(8), 535(37), 419(2), 373(33), 343(100), 297(6)
**39**	44.59	Borapetoside A	C_26_H_3__3_O_12_	[M − H]^−^	537.19604	537.19665	1.136	519(4), 493(2), 491(62), 375(3), 371(5), 357(1), 341(2), 329(100), 297(1)
**40**	45.22	8-Hydroxycolumbin	C_20_H_2__1_O_7_	[M − H]^−^	373.12759	373.12817	1.554	358(2), 343(45), 325(6), 313(100)
**41**	45.78	6-Hydroxycolumbin	C_20_H_2__1_O_7_	[M − H]^−^	373.12759	373.12817	1.554	358(9), 355(7), 343(79), 329(3), 325(15), 313(100), 305(3), 261(2)
**42**	46.04	Boropetoside G	C_27_H_3__7_O_11_	[M − H]^−^	537.23218	537.23303	1.582	518(1), 375(100), 361(9), 360(2), 357(2), 343(2)
**43**	46.82	(4,6,6,9,10,10)-Methyl 9-acetoxy-2-(furan-3-yl)-4a-hydroxy-10b-methyl-4-oxo-6-(((3,5,6)-3,4,5-trihydroxy-6-(hydroxymethyl)tetrahydro-2*H*-pyran-2-yl)oxy)dodec-ahydro-1*H*-benzo[f]isochromene-7-carboxylate *	C_28_H_3__7_O_14_	[M − H]^−^	597.2168	597.21778	1.641	579(23), 565(52), 564(45), 551(27), 529(31), 389(10), 329(100)
**44**	46.89	Tinocrisposide	C_27_H_3__5_O_11_	[M − H]^−^	535.21600	535.21738	2.578	520(2), 516(5), 488(7), 373(100), 359(8), 358(6), 355(3), 341(2)
**45**	49.44	Cordifolide A	C_28_H_3__7_O_12_S	[M − H]^−^	597.19922	597.20002	1.340	579(13), 553(33), 550(53), 533(100), 528(25), 467(43), 466(25), 434(18), 372(32), 359(11)
**46**	49.89	(2*R*,5*R*,6*R*,8*S*,9*S*,10*S*,12*S*)-15,16-Epoxy-2-hydroxy-6-*O*-{β-d-glucopyranosyl-(1→6)-*α*-d-xylopyranosyl}-cleroda-3,13(16),14-trien-17,12-olid-18-oic acid methyl ester	C_32_H_4__3_O_16_	[M − H]^−^	683.25416	683.25456	0.585	665(2), 664(11), 637(2), 615(3), 534(3), 520(100), 502(93), 490(12)
**47**	50.40	3-((6,6,7,10,10,10)-7-Hydroxy-6a,10b-dimethyl-4,12-dioxo-6-(((3,5,6)-3,4,5-trihydroxy-6-(hydroxymethyl)-tetrahydro-2H-pyran-2-yl)oxy)-2,4,4,5,6,6,7,10,10,10-decahydro-1*H*-10,7-(epoxymethano)benzo[f]isochromen-2-yl)tetrahydrofuran-2-yl acetate *	C_28_H_3__7_O_14_	[M − H]^−^	597.21680	597.21778	1.641	579(1), 561(15), 551(9), 495(6), 487(7), 486(66), 435(4), 433(100), 297(44)
**48**	51.12	Tinosposinenside C	C_26_H_3__5_O_12_	[M − H]^−^	539.21155	539.21230	1.391	521(3), 497(100), 479(86), 478(8), 377(2)
**49**	52.20	(1,3,10,10)-9-(2-Methoxy-2,5-dihydrofuran-3-yl)-10a-methyl-4,7-dioxo-3a-(((2,3,4,5,6)-3,4,5-trihydroxy-6-(hydroxymethyl)tetrahydro-2*H*-pyran-2-yl)oxy)tetra-decahydroisobenzofuro[7,1-fg]isochromen-1-yl acetate *	C_28_H_3__7_O_14_	[M − H]^−^	597.21655	597.21778	2.060	579(0.4), 551(98), 509(7), 491(100), 481(6), 435(2)
**50**	59.05	6′-*O*-Lactoylborapetoside B	C_30_H_39_O_14_	[M − H]^−^	623.23434	623.23343	−1.460	513(3), 460(100), 458(7), 446(7), 444(4), 297(1), 283(12)
**51**	59.98	Columbin ^a^	C_20_H_2__1_O_6_	[M − H]^−^	357.13297	357.13326	0.812	
**52**	60.37	Tinospinoside E	C_26_H_29_O_11_	[M − H]^−^	517.16986	517.17043	1.102	499(26), 473(10), 471(20), 381(13), 355(6), 341(11), 162(100), 151(6)
**53**	61.73	Tinosporaside	C_25_H_3__1_O_10_	[M − H]^−^	491.19087	491.19117	0.611	473(28), 472(100), 460(8), 444(0.3), 327(0.4), 312(2)
			C_26_H_3__3_O_12_	[M − H + HCOOH]^−^	537.19697	537.19665	−0.596	519(4), 518(11), 491(25), 490(100), 357(3), 343(4), 327(8)
**54**	62.14	Sagittatayunnanoside B	C_33_H_4__7_O_17_	[M − H]^−^	715.28030	715.28077	0.657	670(2), 628(1), 652(1), 552(100), 551(16)
**55**	63.08	Tinospinoside C	C_27_H_3__5_O_12_	[M − H]^−^	551.21155	551.21230	1.361	533(73), 507(90), 505(100), 483(85), 415(84), 389(22), 343(81)
**56**	63.14	Sagittatayunnanoside C	C_32_H_4__7_O_15_	[M − H]^−^	671.28912	671.29094	2.711	653(1), 627(2), 509(50), 347(27), 329(100), 301(70), 241(25)
**57**	63.83	Tinosponone	C_19_H_2__1_O_5_	[M − H]^−^	329.13791	329.13835	1.337	314(100), 311(24), 285(70), 191(18)
**58**	65.99	Sagittatayunnanoside A	C_26_H_3__7_O_10_	[M − H]^−^	509.23792	509.23812	0.393	491(5), 465(5), 347(33), 329(100), 301(89), 257(9), 241(11)
**59**	69.79	2-*O*-Lactoylborapetoside B	C_30_H_39_O_14_	[M − H]^−^	623.23334	623.23343	0.144	608(1), 591(23), 551(7), 486(16), 460(100), 297(34)
**60**	78.98	Tinotufolin D	C_20_H_2__5_O_4_	[M − H]^−^	329.17441	329.17473	0.972	311(36), 285(100), 293(7), 267(10), 249(16)
**61**	82.48	(2aβ,3*α*,5aβ,6β,7*α*,8a*α*)-6-2-(3-Furanyl)ethyl-2a,3,4,5,5a,6,7,8,8a,8b-decahydro-2a,3-dihydroxy-6,7,8b-trimethyl-2*H*-naphtho1,8-*bc*furan-2-one	C_20_H_2__7_O_5_	[M − H]^−^	347.18491	347.18530	1.123	329(63), 303(3), 301(100), 285(3), 187(1)
**62**	88.91	(3,4,5,8)-Methyl-5-(2-(furan-3-yl)ethyl)-3-hydroxy-5,8a-dimethyl-3,4,4,5,6,7,8,8a-octahydronaphthalene-1-carboxylate *	C_20_H_2__7_O_4_	[M − H]^−^	331.19016	331.19038	0.664	313(9), 287(15), 285(100), 283(30), 271(2), 257(5), 243(2), 237(0.4)
**63**	95.27	Tinotufolin C	C_21_H_3__1_O_5_	[M − H]^−^	363.21662	363.21660	−0.055	345(0.1), 334(0.2), 317(69), 295(100)

^a^ Comparison with standards. * Tentatively identified as new compounds.

## Supplementary Materials

The supplementary files are available online.
