# Supplementary material for: Rapid Screening and Identification of Diterpenoids in Tinospora sinensis Based on High-Performance Liquid Chromatography Coupled with Linear Ion Trap-Orbitrap Mass Spectrometry
Source: Molecules. 2017 May 31;22(6):912. doi: 10.3390/molecules22060912 (PMC6152629; doi:10.3390/molecules22060912)
Supplement: Supplementary file 1 [file molecules-22-00912-s001.pdf]

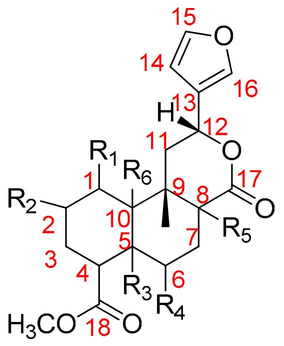

	R1	R2	R3	R4	R5	R6
1	OMe	OMe	β-Me	β-OGlc(Ac)4	β-H	β-H
8	α-OAc	OH	β-H	α-OGlc	H	α-H
25	H	α-OAc	β-H	α-OGlc	β-OH	α-H
43	H	α-OAc	β-H	α-OGlc	α-OH	α-H

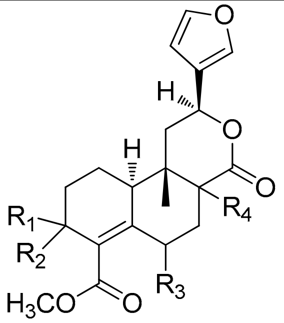

	R1	R2	R3	R4
2	H	H	α-O-β-D-Glc	β-OH
6	α-O-β-D-Glc	β-H	H	β-OH
16	H	H	α-OGlc	α-H

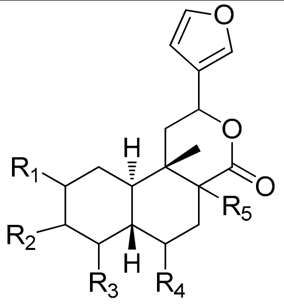

	R1	R2	R3	R4	R5
3	α-OAc	α-OAc	β-OH	α-OGlc	β-H
5	α-OH	α-OH	α-OGlc	H	β-OH
14	α-OAc	α-OAc	β-OH	α-OGlc	α-H
18	α-OH	α-OH	α-OGlc	β-OH	H
20	α-OAc	α-OAc	α-OGlc	H	β-OH

31	*α*-OAc	*α*-OH	*α*-OGlc	H	*β*-OH
38	*α*-OAc	*α*-OAc	*α*-OGlc	H	*β*-H
48	*α*-OAc	*α*-OH	*α*-OGlc	H	*β*-H

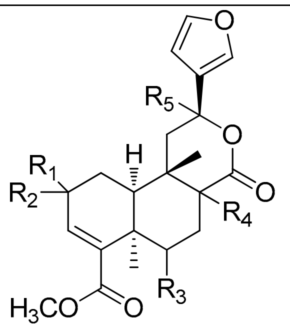

	R1	R2	R3	R4	R5
4	*β*-H	*α*-OH	*β*-O-{*β*-D-Glc-(1→6)-*α*-D-xylo}	H	*α*-H
9	H	*α*-OH	*β*-O-(*β*-D-Glc)	*α*-H	H
11	H	*α*-OGlc	H	*β*-OH	*α*-H
19	*β*-H	*α*-OH	*β*-O-(*β*-D-Glc)	*β*-H	*α*-H
27	H	*α*-OGlc	*α*-OGlc	*α*-H	H
28		=O	*β*-O-(*β*-D-Glc)	*α*-H	*α*-H
36	*β*-H	α-H	β-O-(*β*-D-Glc)	*β*-H	*α*-H
44	H	H	*β*-OGlc	H	*α*-H
46	*β*-H	*α*-OH	*β*-O-{*β*-D-xylo-(1→6)-*α*-D-Glc}	H	*α*-H
50	H	*α*-OH	*α*-OGlc(lactoyl)	*α*-H	H
59	H	*α*-O-lactoyl	*α*-OGlc	*α*-H	H

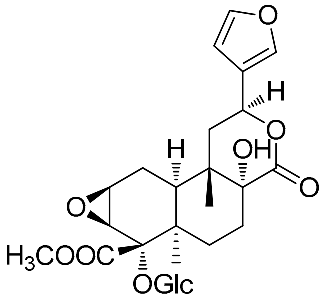
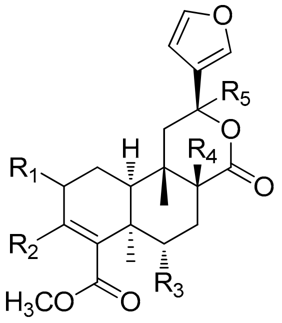
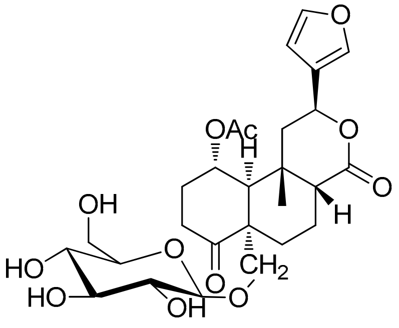

7 13

	R1	R2	R3	R4	R5
10	H	OH	OGlc	*β*-OH	H
55	*α*-OGlc	H	H	*α*-OH	*α*-H

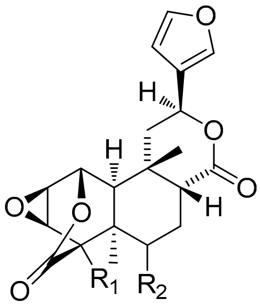 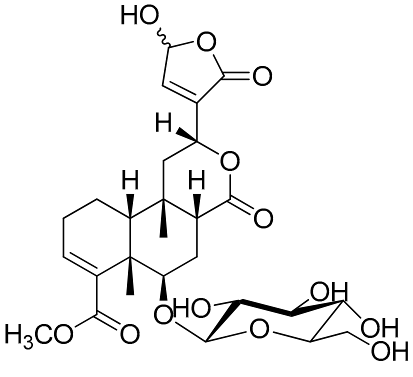 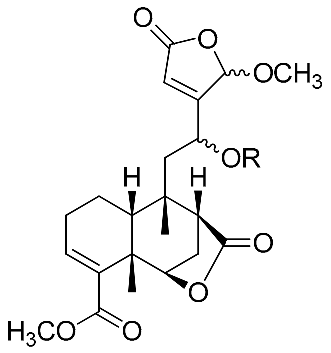

15 17 R=β-D-Glc

	R_1	R_2
12	H	α-O-(β-D-Glc)
21	α-O-(β-D-Glc)	H

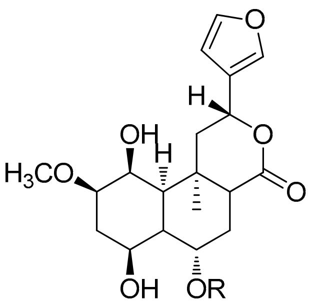 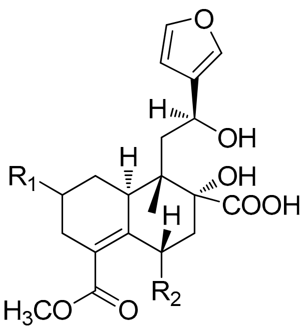 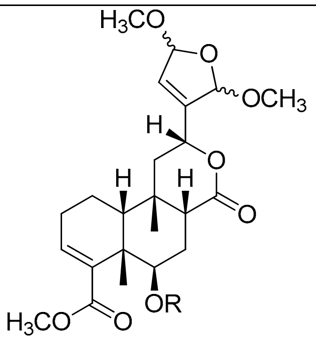

22 R=β-D-Glc 24 R=β-D-Glc(Ac)$_4$

	R_1	R_2
23	H	α-O-β-D-Glc
37	α-O-β-D-Glc	H

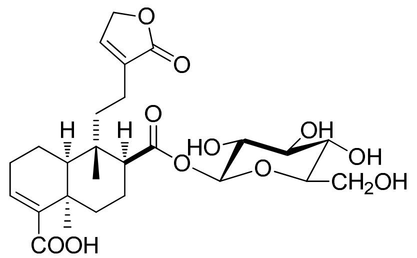 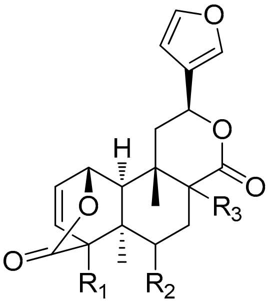 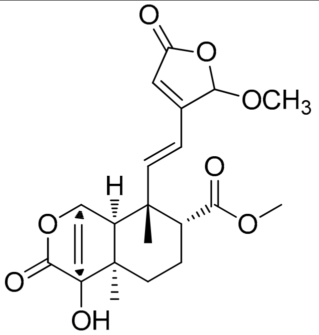

26 32

	R_1	R_2	R_3
29	β-OH	H	H
30	α-OGlc	H	α-H
40	α-OH	H	β-OH
41	OH	α-OH	β-H
51	α-OH	H	β-H

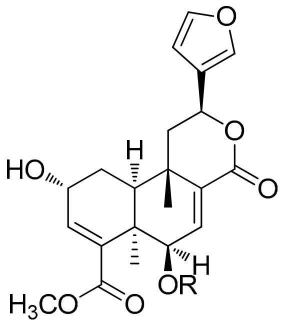

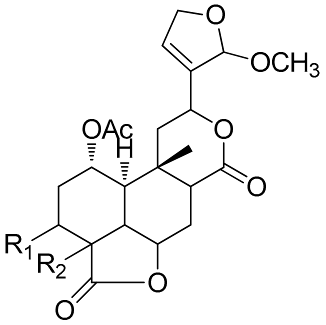

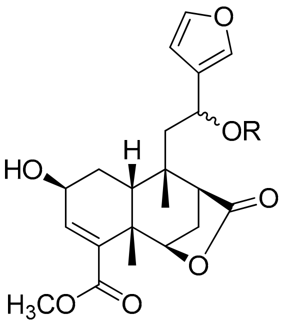

33 R=β-D-Glc

35 R=β-Glc

	R_1	R_2
34	α-OGlc	H
49	H	α-OGlc

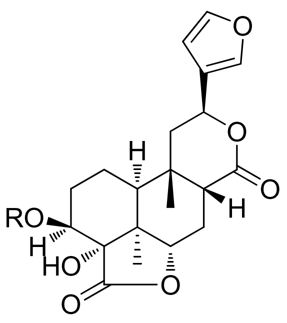

39 R=β-D-Glc

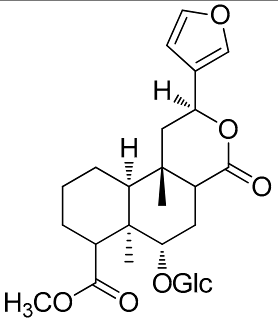

42

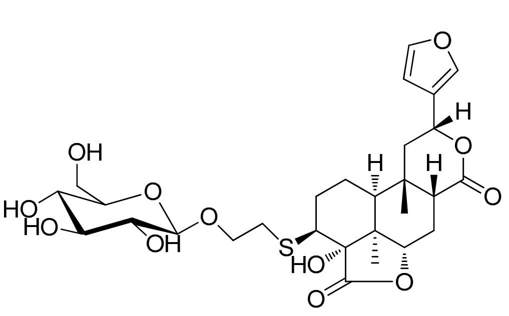

45

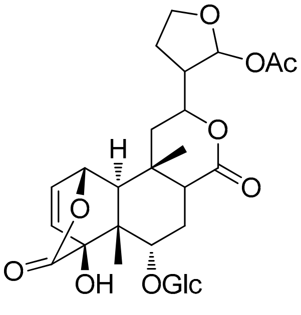

47

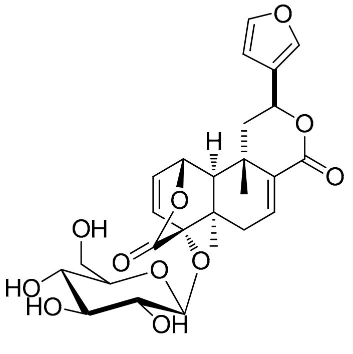

52

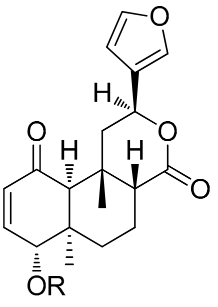

53 R=β-D-Glc

57 R=H

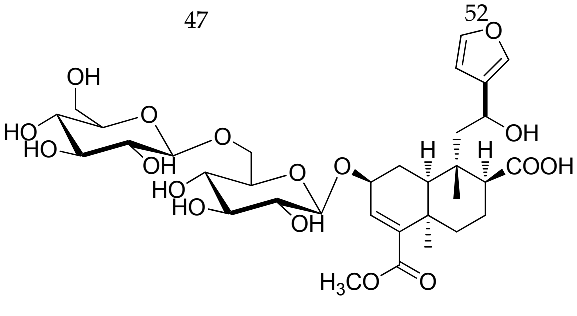

54

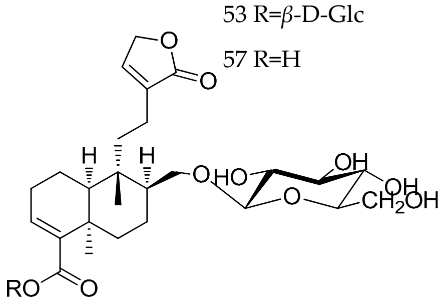

56 R=Glc

58 R=H

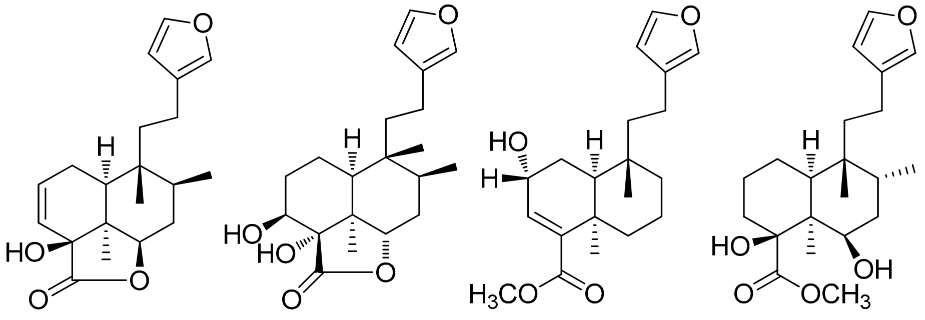

60 61 62 63

Figure A. The structure of chemical constituents identified in *Tinospora sinensis* by HPLC-LTQ-Orbitrap

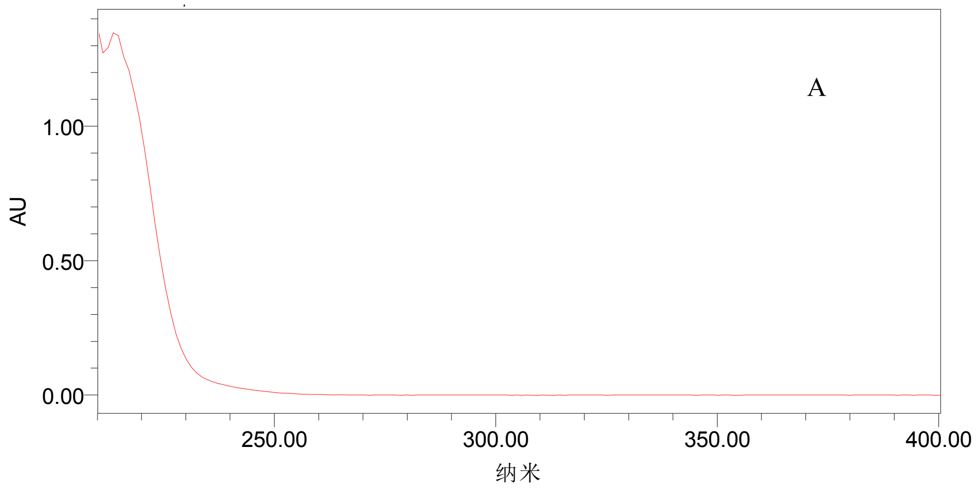

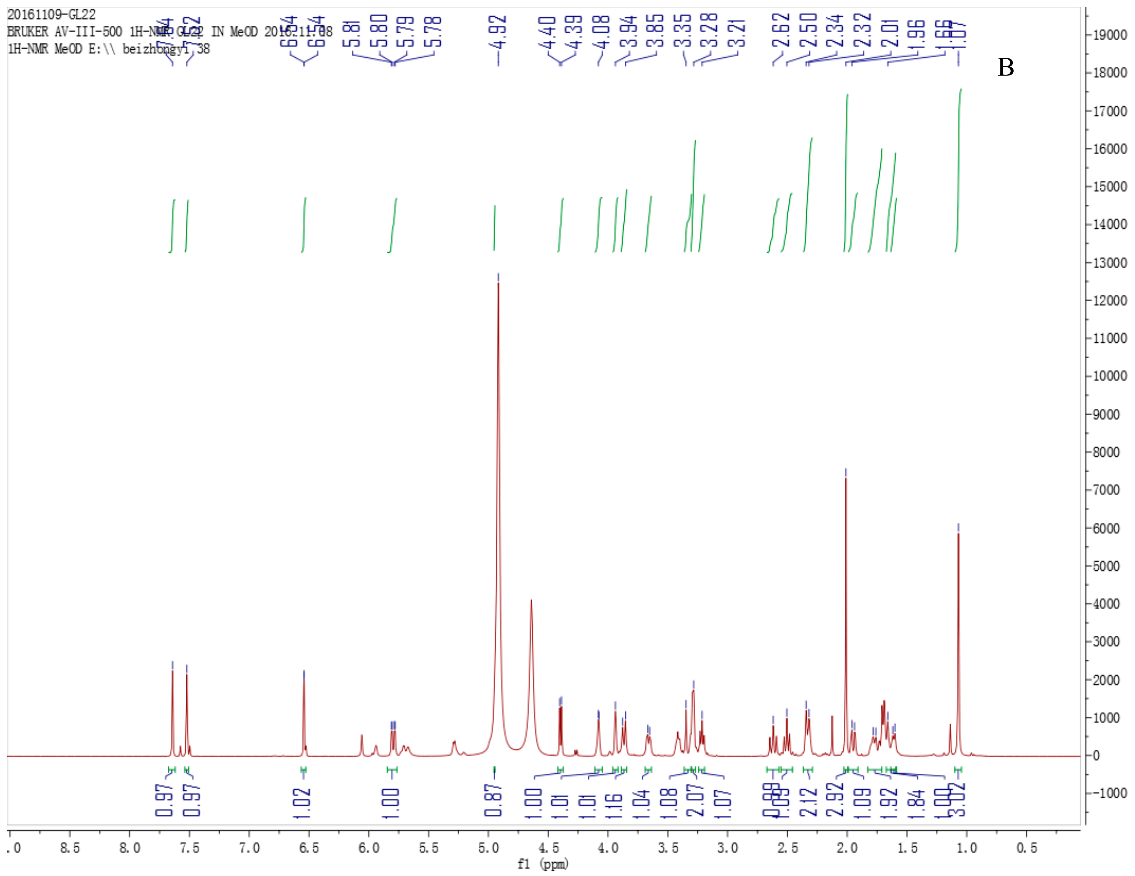

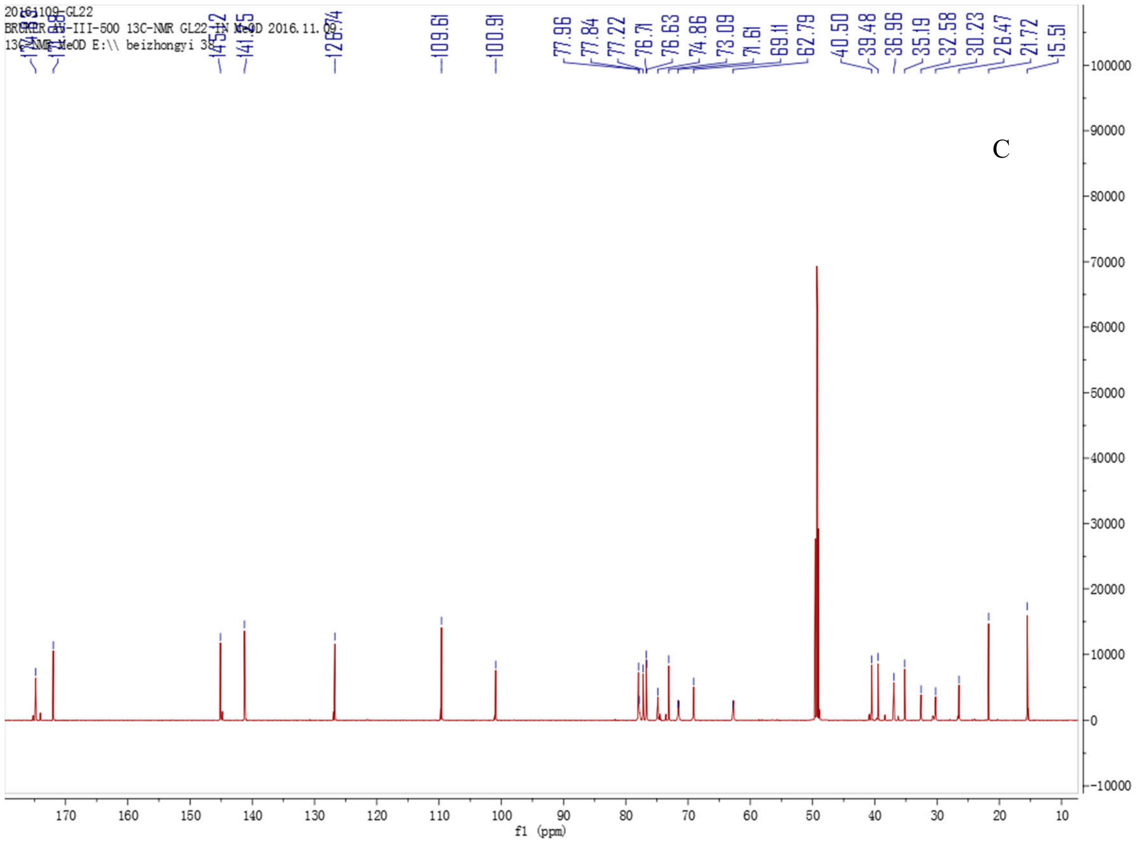

Figure B. A UV figure for Tinosineside A; **B** ^{1}H NMR of Tinosineside A; **C** ^{13}C NMR of Tinosineside A

Table A. ^{1}H-(500MHz) NMR spectral data of Tinosineside A

No.	^{1}H-NMR	Reference data
1	4.93（1H，dd，J=11.0,2.4Hz）	4.93（1H，dd，J=11.2,2.8Hz）
2	4.08（1H，brd，J=2.4Hz）	4.07（1H，brd，J=2.8Hz）
3a	1.66（1H，m）	1.68（1H，m）
3b	2.33（1H，m）	2.33（1H，m）
4	3.94（1H，brs）	3.93（1H，brs）
5	1.77（1H，m）	1.77（1H，m）
6a	1.60（1H，s）	1.60（1H，s）
6b	1.77（1H，m）	1.77（1H，m）
7a	1.66（1H，m）	1.68（1H，m）
7b	2.33（1H，m）	2.33（1H，m）
10	2.50（1H，dd，J=11.0,11.1Hz）	2.53（1H，dd，J=11.2,10.0Hz）
11a	1.95（1H，dd，J=14.4,3.3Hz）	1.97（1H，dd，J=14.2,3.7Hz）
11b	2.62（1H，dd，J=14.4,12.6Hz）	2.63（1H，dd，J=14.2,12.6Hz）
12	5.79（1H，dd，J=12.6,3.4Hz）	5.78（1H，dd，J=12.6,3.7Hz）
14	6.54（1H，d，J=1.0Hz）	6.52（1H，dd，J=1.2,0.7Hz）

15	7.52（1H，m）	7.49（1H，m）
16	7.64（1H，brs）	7.60（1H，d，J=0.7Hz）
20	1.07（3H，s）	1.07（3H，s）
COCH3	2.01（3H，s）	1.99（3H，s）
1'	4.39（1H，d，J=7.8Hz）	4.37（1H，d，J=7.8Hz）
2'	3.21（1H，dd，J=8.3,7.8Hz）	3.19（1H，dd，J=9.2,7.8Hz）
3'	3.35（1H，dd，J=8.3,5.6Hz）	3.37（1H，dd，J=9.2,9.2Hz）
4'	3.28（1H，dd，J=5.6,8.6Hz）	3.27（1H，dd，J=9.2,9.2Hz）
5'	3.24（1H，m）	3.25（1H，m）
6'a	3.66（1H，dd，J=11.7,7.5Hz）	3.64（1H，dd，J=11.9,5.5Hz）
6'b	3.86（1H，dd，J=11.7,1.6Hz）	3.84（1H，dd，J=11.9,1.9Hz）

Table B. ^{13}C-(500MHz) NMR spectral data of Tinosineside A

No.	^{13}C-NMR	Reference data	No.	^{13}C-NMR	Reference data
1	77.2	77.4	14	109.6	109.6
2	69.1	69.3	15	145.1	145.0
3	32.6	32.8	16	141.3	141.3
4	76.7	76.8	17	174.8	174.8
5	39.5	39.9	20	15.5	15.5
6	26.5	26.6	$\underline{CO}CH_3$	172.0	171.6
7	30.2	30.5	$CO\underline{CH_3}$	21.8	21.6
8	76.6	76.7	Glc-1'	100.9	101.2
9	40.5	40.7	-2'	74.9	75.1
10	35.2	35.4	-3'	78.0	78.2
11	37.0	37.2	-4'	71.6	71.9
12	73.1	73.2	-5'	77.8	78.2
13	126.7	127.1	-6'	62.8	63.1
